# sunTILL: a TILLING resource for gene function analysis in sunflower

**DOI:** 10.1186/1746-4811-7-20

**Published:** 2011-06-30

**Authors:** Wilma Sabetta, Vittorio Alba, Antonio Blanco, Cinzia Montemurro

**Affiliations:** 1 Department of Agro-Forestry and Environmental Biology and Chemistry, Section of Genetics and Breeding, University of Bari, via Amendola 165/A, 70126, Bari, Italy

## Abstract

**Background:**

Cultivated sunflower (*Helianthus annus *L.) is a globally important oilseed crop, subjected to intensive genetic and genomic studies. Although classical mutagenesis has successfully been applied to *Helianthus *genus in the past, we have developed the first sunflower TILLING resource.

**Results:**

To balance the maximum mutation density with an acceptable plant survival rate, a 'kill curve' analysis was first conducted with different ethylmethanesulfonate (EMS) dosages and different exposure times. According to the germination rate, a treatment with 0.7% EMS for 6 h was chosen. An M_2 _progeny of 3,651 fertile plants was obtained. Totally, 4.79% of the whole population showed clear aberrant phenotypes. A microsatellite analysis on a representative sample of the original seed stock and mutant lines confirmed the uniformity of the genetic background of plant material. The TILLING procedure was successfully applied to sunflower genome, initially by a *Cel*I-nuclease mismatch cleavage assay coupled with a DNA-pooling level test. To investigate the efficiency of the mutagenic treatment, a pilot screening was carried out on 1,152 M_2_ lines focusing on four genes, three involved in the fatty acid biosynthetic pathway and one for downy mildew resistance. A total of 9 mutant lines were identified and confirmed by sequencing; thereby, the estimated overall mutation frequency for the pilot assay resulted to be 1/475 kb.

**Conclusion:**

A first TILLING population for a high throughput identification of EMS-induced point mutations in sunflower genome has been successfully obtained. This represents a powerful tool to a better understanding of gene function in sunflower.

## Background

Cultivated sunflower (*Helianthus annuus *L.) is a globally important oilseed crop subjected to intensive molecular genetic and genomic studies during recent decades [1]. Sunflower belongs to the genus *Helianthus*, which is native to temperate areas of North America and includes 12 annual and 37 perennial species [2,3]. The world production of sunflower is estimated to be 23.4 million tons [4]; seed production in the world increased by 24% (or 5 million metric tons) between 1993 and 2003 [5]. After maize, it is the second largest hybrid crop, and the fifth largest among the oilseed crops, after soybean, rapeseed, cottonseed, and groundnut. The largest producers of sunflower seeds are Russia and Ukraine, followed by the European Union with 4.9 million tons [4], where this crop is mainly cultivated in France, Hungary, Italy, and Spain, thanks to their ideal climate. Sunflower can be used for different purposes: as an oilseed crop, edible confection, birdseed and, to a much lesser extent, as an ornamental for home gardens and the cut-flower industry.

Wild species of sunflower are characterized by a high genetic diversity as a consequence of their adaptation to a wide range of environments [3]. They harbour a significant variability with respect to a number of traits such as disease and pest resistance, quality and composition of seed compounds. The genetic polymorphism of cultivated sunflower is reduced to 40-50% of the diversity observed in the wild germplasm as a result of domestication [6]. The development of new genetic variability in sunflower is aimed at producing a source of agriculturally useful alleles or new genotypes.

Many efforts in sunflower plant breeding have been proposed for the improvement of desirable traits. Genetic engineering [7], traditional breeding approaches [1], *in vitro *breeding techniques (somaclonal variation) and conventional mutation technologies [8] have been used to improve yield, oil quality and disease-, salt- and pest-resistance of the sunflower crop. TILLING (Targeting Induced Local Lesions IN Genomes) is a reverse genetic technique that is suitable for most plants [9]. In a TILLING project, the chemical mutagenic treatment providing an easy and cost-effective way to saturate a genome is coupled with a PCR-based mutation detection. By using alkylating agents such as ethylmethanesulfonate (EMS) that cause random point mutations at high density, an allelic series of missense mutations can be discovered by TILLING; short insertion/deletions (INDELs) are reported to be detected by this technology too [10]. Thus, with only a small population, multiple alleles of a specific gene may be obtained regardless of the gene size [11]. Gene regions are targeted for mutation discovery, using PCR and standard SNP (single nucleotide polymorphism) discovery methods. The success of the TILLING approach relies on the construction of high quality DNA mutant libraries, in which DNA sampling and preparation are the most critical steps. An accurate evaluation of the genetic uniformity both of seed stock used for the mutagenic treatment and of the mutagenized material is extremely important. As reported by Wu *et al*. [12], genotyping plant material by means of microsatellite markers (SSR) can be an efficient tool to verify the uniformity and purity of the seed stock used in a TILLING project.

Although classical mutagenesis has been successfully employed in sunflower breeding programs over the last 40 years, sunflower TILLING resources have not been reported yet. The objectives of the present study were: 1) the development of the first sunflower TILLING population, where single nucleotide lesions are induced by conventional EMS mutagenesis; 2) the optimization of TILLING procedure for an efficient sunflower SNP detection system and the mutation density evaluation by screening a subset of M_2 _lines; 3) the phenotyping of the M_2 _generation for the presence of mutations in comparison to the untreated sunflower.

## Methods

### Plant material and EMS mutagenesis

Seeds from the inbred line *GV342 *of cultivated sunflower (*Helianthus annus*) were used for the mutagenesis experiment. To test the 'kill curve', eight batches of 100 seeds each were treated with different EMS concentrations (0.5, 0.7, 1.0 and 1.5%); all concentrations were also tested for two different exposure times (3 and 6 h) at 20°C and applying a gentle shaking. Then seeds were washed with tap water three times for 5 min each and a fourth time for 30 min. Subsequently, they were transferred on wet Whatman paper in Petri dishes in a growth chamber at 20°C and 8 h photoperiod.

Based on the percentage germination, the 6 h-treatment with 0.7% EMS was chosen to mutagenize a batch of ~30,000 seeds. After an overnight air-drying on Whatman paper at room temperature, seeds were mechanically sown in the experimental field close to Bari (Southern Italy) with a cultivation distance between and within rows of 70 and 30 cm respectively. Fertilization and herbicidal treatments were applied once and the field was regularly watered. To allow self-pollination, the floral bottom of each M_1 _plant was covered with a TNT bag before anthesis. At physiological maturity, the main head from each individual plant was harvested and M_2 _seeds were collected and stored at 4°C.

Four to ten M_2 _seeds for each family were sowed in the field using the same conditions as for the M_1 _generation. At the 3-4 leaf stage, most of the emerged plants were manually thinned to have one plant per family. M_2 _plants were regularly monitored for the presence of morphological mutants, using the untreated plants as reference, and forced to self-pollinate by bagging the inflorescences. Germination and sterility rates of the M_2 _generation were also evaluated. Based on a visual characterization of plants at the key development stages, from germination to maturity, a systematic phenotypic scoring of the mutant population was carried out. Seven main traits were chosen from the worldwide sunflower descriptor list promoted by the *International Board for Plant Genetic Resources *[13]. Phenotypes were organized into the following categories: leaf colour, shape and size; internode length; type of branching; venation colour and seed colour. Phenotypic alterations were annotated under field-growing conditions and referred to untreated plants; a detailed photographic documentation was also collected. M_3 _seeds from individual plants were harvested, aired and vacuum-stored at 4°C.

### DNA extraction and normalization

Leaf material from each M_2 _plant was collected for DNA isolation. Approximately 100 mg of lyophilised mature leaves were located in 2 ml single tubes and crashed using a Mixer-Mill (Retsch-Muhle MM30, Qiagen, Germany) two times for 45 sec at 30 Hz with two 3.175/III-mm-diameter inox spheres. DNA was extracted according to Li *et al*. [14] with one modification: all the centrifugations in the second part of the protocol were elongated up to 2-5 min. Genomic DNA concentration and quality were measured both by means of a NanoDrop™ 1000 Spectrophotometer (Thermo Scientific, USA) and 1% agarose gel. DNAs were transferred into 96-well plates and normalized to a standard concentration of 20 ng/μl by adding 0.1 × TE buffer (10 mM Tris-HCl pH 8.0 and 1 mM EDTA).

### SSR analysis

The genetic uniformity and purity of *GV342 *seed stock were checked by microsatellite markers. DNAs from 96 wild-type samples, 96 randomly chosen mutant lines, five commercial hybrids from the SIS-Società Italiana Sementi (San Lazzaro, Bologna, Italy) and five experimental hybrids kindly provided by the University of Udine (Italy), were genotyped with 15 SSR: ORS342, ORS380, ORS386, ORS407, ORS486, ORS767, ORS780, ORS878, ORS938, ORS959, ORS966, ORS995, ORS1013, ORS1112, ORS1114 [15,16]. SSR primer sequences, linkage group, fragment size and annealing temperature are reported in Table 1. PCR mixture was carried out with 40 ng of genomic DNA, 1× Buffer (10 mM Tris-HCl, pH 8.3, 10 mM KCl), 200 μM each dNTPs, 3.0 mM MgCl_2_, 0.25 μM each microsatellite primer, and 0.06 U Taq polymerase (Euroclone) in a final volume of 25 μl. Forward primers of ORS486, ORS767 and ORS938 microsatellites were fluorescently labelled with 6-FAM or 8-HEX (Sigma). The amplification program was as following: 5 min at 95°C; 35 cycles at 95°C for 30 sec, 45 sec at the primer-specific annealing temperature, 1 min at 72°C; a final extension at 72°C for 20 min. PCR products were subjected to electrophoresis on 2.5% SeaKem^® ^LE Agarose gel (Lonza, Switzerland), while the labelled amplicons were detected by capillary electrophoresis using an ABI PRISM 3100 Avant Genetic Analyzer (Applied Biosystem, USA) and analysed using GeneMapper v3.5 genotyping software. The internal molecular weight standard was 500-ROX (Applied Biosystem, USA).

**Table 1:** Genetic uniformity analysis by microsatellite markers.

SSR marker	Linkage group **(ref. **[16]**)**	Repeat motif	Annealing temperature	Detected alleles (bp)		
				
				hybrids	wildtype	M_2 _lines
ORS 959	I	^(GT)^7	60°	245 - 255	245	245
ORS 342	II	^(GT)^10	58°	320 - 340	340	340
ORS 486	II	^(AC)^11	55°	126 - 130 - 140	130	130
ORS 1112	III	^(AG)^15	60°	365 - 370 - 380	380	380
ORS 1114	III	^(CT)^13	60°	250 - 260 - 270	250	250
ORS 966	VII	^(GT)^9	60°	380 - 395 - 410	380	380
ORS 780	VIII	^(AG)^28	58°	295 - 320	295	295
ORS 1013	VIII	^(CT)^14	60°	190 - 200	200	200
ORS 938	IX	^(GT)^20	55°	311 - 313 - 315 - 320	315	315
ORS 380	X	^(GT)^9	58°	405 - 425 - 460	405	405
ORS 878	X	^(AC)^11	60°	200 - 220 - 235	200	200
ORS 767	XII	^(GT)^7^(GA)^16	52°	368 - 370	368	368
ORS 995	XIII	^(CT)^29	60°	240 - 250	240	240
ORS 407	XVI	^(GT)^13	58°	440 - 450 - 460	450	450
ORS 386	XVII	^(GT)^20	55°	290 - 310	310	310

Linkage group, repeat motif, annealing temperature, number and size of alleles detected in ten commercial and experimental hybrids and in a sub-set of untreated samples and mutant lines are listed per each SSR markers.

### SNP detection test

Two sunflower genotypes (i.e. *XRQ *and *PSC8 *from INRA, France), known to be polymorphic for a single nucleotide at one specific locus (Dr. P. Vicourt, personal communication), were used as positive control to optimize the sunflower SNP detection system. Two different tests were carried out: a *Cel*I-nuclease mismatch cleavage assay and a DNA pooling test. In the first test, DNAs from *XRQ *and *PSC8 *genotypes were mixed together in the same proportions to create two different 2-fold pools, that were used as template for the amplification and the subsequent preliminary digestion test. Once optimized the *Cel*I restriction analysis, the best enzymatic conditions were used for the DNA pooling test, which was performed by the combination of DNAs from *XRQ *and *PSC8 *genotypes in different proportions simulating 2-, 4-, 8-, 12-, 16-, 20-fold pools. Forward and reverse primers were 5'-end labelled with IRDy671 and IRDy781 dyes respectively (Eurofins MWG Operon, Germany). Mixtures of 3:2 (labelled:unlabelled) ratio for the forward primers and 3:1 (labelled:unlabelled) ratio for the reverse primers were used in the PCR reaction. PCR amplification was carried out in a volume of 20 μl containing 20 ng/μl of pooled DNA, 1 × Phusion HF Buffer (Finnzymes, Finland), 0.2 mM each dNTP, 3% DMSO, 0.3 μM each primer and 0.015 U Phusion High-Fidelity DNA Polymerase (Finnzymes, Finland). The PCR was performed in a thermal cycler (Applied Biosystems 9800 Fast Thermal Cycler) using the following cycling program: 98°C for 30 sec; 35 cycles at 98°C for 10 sec, 52°C for 30 sec, 72°C for 30 sec; then a final extension at 72°C for 10 min. The amplification step was followed by the heteroduplex formation: inactivation at 99°C for 10 min; 23 cycles of re-annealing process for 20 sec at 70°C to 69.4°C, decrementing 0.9°C per cycling [17].

### Amplification of candidate genes

The sequences of *kasII, kasIII, fad2-1 *and *AY490791 *genes were identified on SRS-EMBL, NCBI, CGP, TIGR and HeliGene databases and subjected to bioinformatic analysis by CODDLE (http://www.proweb.org/input/) and SoftBerry (http://linux1.softberry.com/berry.phtml) software to predict the gene structures. All the primer pairs were set by using Primer3 (http://frodo.wi.mit.edu/primer3/) and OligoExplorer v. 1.2 programs. PCR reactions were performed in a thermal cycler (Applied Biosystems 9800 Fast Thermal Cycler) using 96-well microtiter plates and carried out in a 20 μl volume consisting of dH_2_O, 1 × Phusion HF Buffer, 0.2 mM each dNTP, 3% DMSO, 0.3 μM each primer (with a ratio of 3:2 and 3:1 labelled:unlabeled for forward and reverse primer, respectively) (Eurofins MWG Operon, Germany), 0.015 U Phusion High-Fidelity DNA Polymerase (Finnzymes, Finland). The thermocycling conditions were 98°C for 30 sec for initial denaturation, followed by 30 cycles at 98°C for 10 sec, an annealing temperature specific for each primer pair for 30 sec, 72°C for 40 sec, one cycle at 72°C for 10 min and 4°C hold for storage. Five μl of each amplified product were electrophoresed on 1% SeaKem^® ^LE Agarose gel (Lonza, Switzerland) to verify the PCR efficiency and concentration before the subsequent TILLING step.

### *Cel*I nuclease mismatch cleavage assay and purification step

The preliminary *Cel*I digestion test was carried out using progressive enzyme dilutions and different reaction times. Ten μl of amplification product were incubated for different times (15, 30 and 45 min) at 45°C with differentially diluted (1:2, 1:4, 1:10 and 1:20) Surveyor *Cel*I enzyme (Transgenomics, Omaha, USA) in a 10 × Buffer (10 mM HEPES, pH 7.0, 10 mM KCl, 10 mM MgCl_4 _7H_2_0, 0.002% Triton X-100 and 10 μg/ml bovine serum albumen). *Cel*I digestion was stopped by adding 5 μl of 75 mM EDTA (pH 8.0) and freezing samples (-20°C) for at least 3 h. For the pilot screening, a 1:20 enzyme dilution for 45 min reaction was always used as standard conditions.

Sample purification was performed by precipitation with 5 μl 3 M Na-Acetate (pH 5.2), 75 μl 99.8% EtOH and 10 μl dH_2_O. After shaking for 15-20 min, the samples were centrifuged at 4,500 rpm for 30 min at 20°C; the supernatant was carefully discarded. Then plates were spin upside down on a filter paper up to 500 rpm, washed with 100 μl 75% EtOH and re-centrifuged at 4,500 rpm for 30 min at 20°C. Again, the supernatant was discarded on filter paper and plates were spin facedown on a filter paper up to 500 rpm. Then, samples were dried for 20 min at room temperature and resuspended in 8 μl formamide loading buffer (33% deionised formamide, 25 mM Tris pH 7.5, 25 mM EDTA and ~0.02% bromophenol blue) by shaking (300 rpm) for 5-10 min.

### Li-COR gel electrophoresis

Samples were denatured by incubation at 95°C for 5 min, placed on ice, loaded on gel by a 100 teeth paper comb and finally electrophoresed on Li-COR DNA Analyser. Electrophoresis was performed through a 6.0% LongRanger^® ^polyacrylamide (Biozym, FMC Corporation, Austria), 7 M urea gel in 1 × Tris-Borate-EDTA running buffer. The laser focusing and gel warming to 50°C were obtained by a pre-run at 1.500 V, 40 mA and 40 W for 20 min. Gel images were analysed visually for the presence of cleavage products using GelBuddy (http://www.proweb.org/gelbuddy) [18] and Adobe Photoshop (Adobe System Inc., CA, USA) software.

To estimate the mutation frequency for each screened gene, the total number of analysed base pairs (obtained multiplying the number of screened plants per the fragment size) was divided by the number of identified mutations. Because of priming and PCR artefacts on the top and the bottom of LiCOR gels, the detection of mutations in the terminal 50 bp at each end of the amplicons was difficult and for this reason 100 bp were subtracted from the size of each amplicon to obtain the effective screened window size.

## Results

### Production of a sunflower mutant population

In the past, a broad spectrum of genetic resources was developed for cultivated sunflower and most of the available germplasm has been accurately characterized by molecular markers [1]. Information about genetic diversity [19-21], molecular mapping [15,22], gene mapping [23-26], construction of cDNA, BAC and BIBAC libraries [27,28] are currently available. Many examples of successfully applied traditional mutagenesis have been reported [8,29,30], but no sunflower TILLING resource has previously been developed.

The inbred line *GV342 *was chosen for the development of the sunflower TILLING platform. To balance the maximum mutation density with an acceptable plant survival rate, a 'kill curve' analysis was first conducted on batches of 100 seeds treated with different EMS dosages, ranging from 0.5 to 1.5% and two different exposure times (3 and 6 h) (Table 2). Seeds treated with 0.5 and 0.7% EMS for 3 h showed only slight differences in the germination rate (78 and 75% respectively) in comparison with the control (untreated seeds), while a drastic decrease in germinability was observed for seeds treated with higher EMS concentrations. Similar results were obtained in the experiment carried out for 6 h: the observed germination rates unambiguously revealed a robust decrease from the lowest EMS concentration to the highest one. Thirty-one and 45% of germinability were observed for 1.5 and 1.0% EMS dosages; 65% of seeds treated with 0.7% EMS was able to germinate, while the germination rate of plants treated with the lowest dose (0.5% EMS) was slightly reduced in comparison with what obtained in 3-h treatment. Moreover, in both experiments, an untreated seed batch (no EMS) was included, revealing a low decrement of the endogenous seed vitality. On the basis of these results and according to the differences of germination rates for 3- and 6-h treatments at 0.7% EMS, the LD_35 _(lethal dose causing 35% reduction in seed germination) was selected for the 6 h-EMS mutagenic treatment to obtain relatively high mutation density without excessively compromising plant fertility.

**Table 2:** Kill curve analysis.

Exposure time (h)	EMS concentration (%)				
	
	0.0	0.5	0.7	1.0	1.5
**3**	85	78	75	58	40
**6**	86	73	65	45	31

Germination rate (%) of M_1 _sunflower seeds treated with different EMS concentrations (0.5, 0.7, 1.0 and 1.5%) and two exposure times (3 and 6 h). Results refer to 100 seed batches for each treatment.

Thirty thousand M_0 _seeds were mutagenized with 0.7% EMS for 6 h and grown in field conditions. About 13,000 M_1 _plants were obtained, but only 50% of them reached the complete maturity and set seeds. Four to 10 M_2 _seeds per each family were sowed in the field and, at the stage of 3-4 leaves, most of the emerged plants were manually thinned to leave only one M_2 _plant per family. About 64% of the sown seeds were able to germinate: thereby, 4,211 M_2 _plants were obtained and regularly monitored for the presence of phenotypic variations in comparison with the untreated wild-type. Finally, since M_3 _seeds were harvested from 86.7% of fertile plants, an M_2 _population of 3,651 lines was used for leaf DNA sampling and M_3 _seed stocking.

### Phenotypic screening

During the plant cycle, M_2 _generation was assessed for morphological changes at the phenotypic level, such as chlorosis, dwarfism, branching injuries, necrosis, plant sterility or alterations in the leaf or plant morphology. The phenotypic scoring was based on the observation of each plant, using the untreated sunflower phenotype as a reference. According to the worldwide descriptor list promoted by the *International Board for Plant Genetic Resources *[13], seven main phenotypic traits were chosen: leaf colour, size and shape; internode length; presence/type of branching; venation colour; seed colour (Table 3). A detailed photographic documentation for each phenotypic category was collected.

**Table 3:** Phenotypic classes observed by the screening of M_2 _generation.

Phenotype		Mutants	
Major category	Sub-category	(N.)	(%)
1. Leaf colour	Chlorotic	14	0.38
	Light green	43	1.38
	Medium green*	3583	98.14
	Dark green	11	0.30

2. Leaf size	Extremely small	13	0.36
	Medium*	3630	99.42
	Extra large	8	0.22

3. Leaf shape	Oblonge	2	0.05
	Lanceolate	15	0.41
	Triangular	10	0.27
	Cordate*	3609	99.85
	Rounded	2	0.05
	Irregular	13	0.26

4. Internode length	Dwarf	11	0.30
	Short	105	2.88
	Medium*	3505	96.00
	Long	30	0.82

5. Type of branching	No branching*	3607	98.79
	Basal branching	18	0.49
	Top branching	1	0.03
	Fully branched with central head	10	0.27
	Fully branched without central head	15	0.41

6. Venation colour	Green*	3650	99.97
	Anthocyanic	1	0.03

7. Seed colour	White	8	0.22
	Dark*	3612	98.93
	Anthocyanic	31	0.85

According to the IBPGR Descriptors, the major categories and sub-categories for seven main traits are listed. Per each group, the wild-type phenotype is marked (*). In total 3,651 lines were screened.

**Figure 1:**
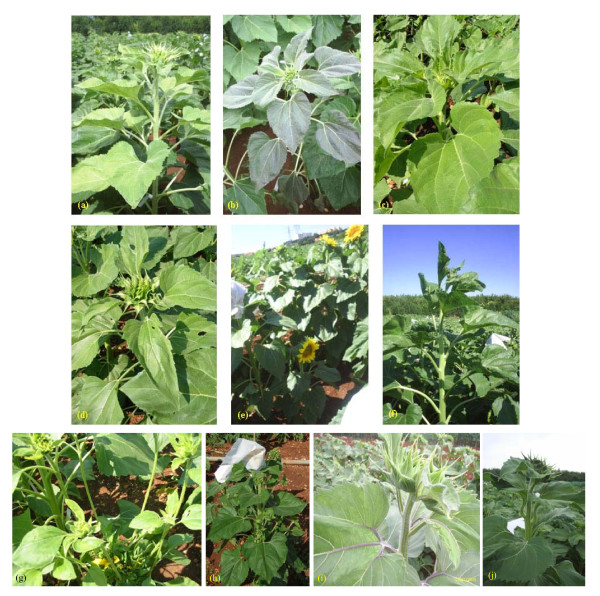
**Selection of morphological mutants observed within the sunflower TILLING population**. The classification refers to the major groups from Table 3. (a) Light and (b) dark green (anthocyanic) coloured leaves; (c) extra large and (d) lanceolate leaves; (e) dwarf plant; (f) mutant with long internode; (g) basal and (h) fully branched plants; (i) mutant with anthocyanic venation; (j) plant with long internodes and deformed stalks.

In total, 68 individuals (1.86% of the M_2 _population) showed anomalous leaf colour (chlorotic, light green and dark green) (Figure 1a and 1b). Concerning the leaf size, 13 and 8 plants showed extremely small and extra large leaves, respectively (Figure 1c), while 42 plants (1.15% of the whole population) showed altered leaf shape traits, such as lanceolate, triangular or irregular leaves (Figure 1d). One plant with deformed petioles was observed and classified as having altered leaf morphology (Figure 1j).

Plants with shorter (Figure 1e) or longer internodes (Figure 1f) than the wild-type in total represented 4% of the population. Regarding the 'plant habit', 98.8% of the population were like the wild-type in not showing lateral shoot branching; only one plant showed top branching, while 18 plants had basal branching (Figure 1g). Ten fully branched plants with the main head (Figure 1h) were observed, while in 15 fully branched plants the main head was missing.

One mutant with an anthocyanic venation colour (Figure 1i) was observed in comparison with the common green colour of the untreated plants. White or anthocyanic coloured seeds, in comparison with the dark wild-type, were grouped in the 'seed colour' category. Eight white and 31 anthocyanic seeds were observed. As a general indication of M_3 _seed yield, only 13.3% of the mutant population resulted sterile and seeds were collected from 3,651 M_2 _plants.

In conclusion, the most common observed phenotypic variation was for plant size (146 plants), followed by leaf colour (68 plants); 42 and 21 plants showed variations in leaf shape and size, respectively. Four distinct branching typologies affected a total of 44 plants, while there were up to 39 individual lines with pale coloured seeds.

In total 175 M_2 _plants, representing 4.8% of the population, showed at least one altered trait. Among these, 138 plants that displayed multiple mutant traits (meaning that they fell into more than one major category) were counted only once. This led to an underestimation of the mutation frequency at the phenotypic level. Moreover, some phenotypic traits (i.e. root or flower characteristics) were not taken into account, therefore the mutation frequency observed in the M_2 _generation may have been underestimated. Detailed morphological and biochemical characterization of larger numbers of M_2 _plants would likely have resulted in the scoring of increased numbers of mutant phenotypes.

### Genetic uniformity analysis

**Figure 2:**
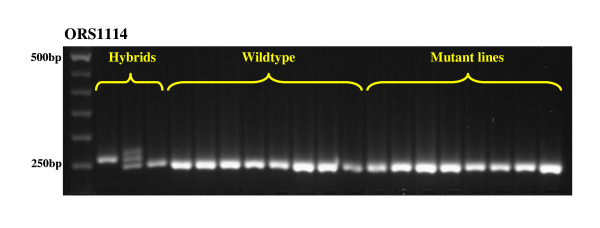
**Genetic uniformity assay by SSR molecular markers**. Electrophoretic pattern of the ORS1114 microsatellite run on 2.5% agarose gel. From left to right, the first lane is represented by the GeneRuler™ 50 bp DNA Ladder Plus (Fermentas); the lanes 2, 3 and 4 represent the molecular profile of three sunflower commercial hybrids, showing the ability of this marker to discriminate the expected polymorphisms. From lane 5 to lane 12 the patterns of some wildtype samples are shown, while the following lanes represent the molecular profiles of a representative sub-set of randomly chosen M_2 _lines.

Sunflower is a predominantly open-pollinated species that completes its reproductive cycle approximately in 6 months. The *GV342 *original seed stock used for the development of our TILLING platform derived from the *HA342 *high oleic acid line (Dr. M. Turi, personal communication). The *GV342 *inbred line was characterized by an oil content of 45-48%, of which 90% is oleic acid. M_1 _plants, obtained from EMS-mutagenized seeds, were obliged to self-fertilize and M_2 _seeds were collected from the main head of each plant. Thereby, a high genetic uniformity of the plant material and a low percentage of heterozygosity were expected. However, due to the predominantly cross-pollinating nature of sunflower and to exclude the possibility of contamination under field conditions, the background uniformity of wild-type and mutant plants was assessed using a set of microsatellite markers distributed overall the sunflower genome. The selected SSR markers (ORS342, ORS380, ORS386, ORS407, ORS486, ORS767, ORS780, ORS878, ORS938, ORS959, ORS966, ORS995, ORS1013, ORS1112, ORS1114) [15,16] are known to reveal a remarkably high level of polymorphism when applied on hybrid cultivars [31] or species and subspecies of sunflower [32]. In the present study, SSR markers were used to genotype five commercial hybrids from the SIS-Società Italiana Sementi (San Lazzaro, Bologna, Italy) and five experimental hybrids from the University of Udine (Italy), to confirm the ability of SSR analysis to identify the expected polymorphisms. Representative samples of the mutant population and the untreated original seed stock were subjected to molecular analysis using the 15 selected SSR markers. The screening was carried out by agarose gel electrophoresis except for three microsatellites (ORS486, ORS767, ORS938), which were fluorescently labelled and detected by capillary electrophoresis. Linkage group, repeat motif and detected alleles on hybrids, wildtype and mutant samples for each SSR marker are reported in Table 1. This analysis confirmed that no cross-pollinations occurred under field condition, since all the analysed samples showed genetic uniformity and the expected microsatellite patterns (Figure 2).

### Optimization of sunflower TILLING procedure

To optimize the TILLING procedure on sunflower genome, two genotypes (i.e. *XRQ *and *PSC8 *from INRA, France), carrying a single nucleotide polymorphism at one specific locus in cluster HuCL00001C250 (http://www.heliagene.org), were used as positive control. These SNP markers allowed the validation of each step of the TILLING procedure.

Two alternative DNA extraction protocols were initially tested. The use of a commercial kit (Nucleospin^®^, Mecherey-Nagel, Germany) provided high quality DNA, but low yields made this protocol unusable for the development of a TILLING platform. Some modifications to the DNA extraction protocol from Li *et al*. [14] as reported in Methods, allowed us to obtain high quality and highly concentrated DNA from both wild-type and mutant samples.

**Figure 3:**
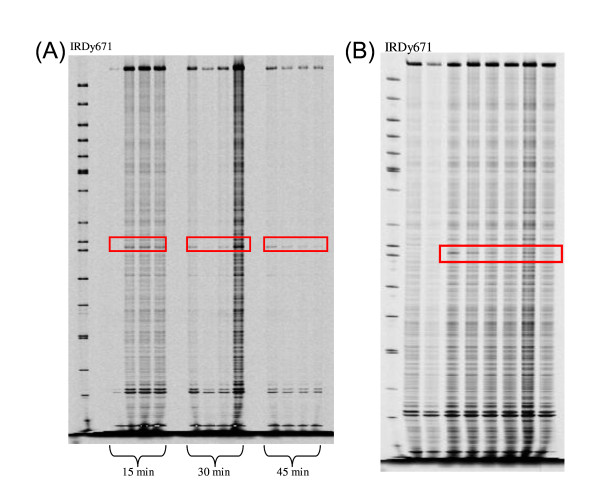
***Cel*I-nuclease mismatch cleavage assay and evaluation of pool capacity for SNP detection. **(A) The IRDy671 image represents reactions carried out for three different digestion times (15, 30 and 45 minutes) with progressive enzyme dilutions (1:2, 1:4, 1:10 and 1:20). The expected identified SNP is highlighted with a red rectangle. The first lane is represented by 50-700 bp Sizing Standard (LiCOR). (B) **Evaluation of pool capacity for *Cel*I SNP detection**. From left to right of the IRDy671 channel image, in the first lane the sizing standard is represented; second and third lanes respectively represent single DNAs from *XRQ *and *PSC8 *genotypes as negative controls; the 2-, 4-, 8-, 12-, 16- and 20-fold pools are shown in the subsequent six lanes.

Since the *Cel*I-based mutation screening was chosen as the detection system of sunflower SNPs, a preliminary *Cel*I-nuclease mismatch cleavage assay, with different enzyme concentrations and different digestion times, was carried out to optimise the reaction conditions. Two-fold pools were generated by mixing equal amounts of DNA from *XRQ *and *PSC8 *genotypes. Progressive *Cel*I dilutions (1:2, 1:4, 1:10, 1:20) were tested and no relevant differences in SNP resolution power were observed, since the expected single polymorphism was clearly detected in both channels (Figure 3A). Moreover, every dilution test was carried out at different digestion times (15, 30 and 45 min): in this case, a progressive reduction of the background and a higher SNP detection power were obtained by the extension of the reaction time from 15 to 45 min (Figure 3A). Thereby, the lowest *Cel*I amount combined with the longest digestion time was the experimental condition chosen for the following test.

Since the efficiency of heteroduplex detection could be influenced by the pool size in terms of DNA amount [10,33], a DNA pooling assay was performed. DNAs from *XRQ *and *PSC8 *genotypes were mixed together in different proportions, simulating 2-, 4-, 8-, 12-, 16- and 20-fold pools. As expected, a progressive reduction of the SNP detection was observed as the pool level increased (Figure 3B): even though the cleaved products were still visible up to the pool ratio of 1:19, the 8-fold pooling size in a two-dimensional format was chosen for the M_2 _DNAs arrangement.

### Candidate genes and reverse genetic pilot screening

To estimate the efficiency of the EMS treatment and to evaluate the mutation frequency, a pilot assay was initially performed on DNAs from 1,152 M_2 _lines, arranged in a two dimensional 8-fold pooling scheme. In particular, four candidate genes were selected: the *kasII, kasIII*, *fad2-1 *and *AY490791 *genes.

**Figure 4:**
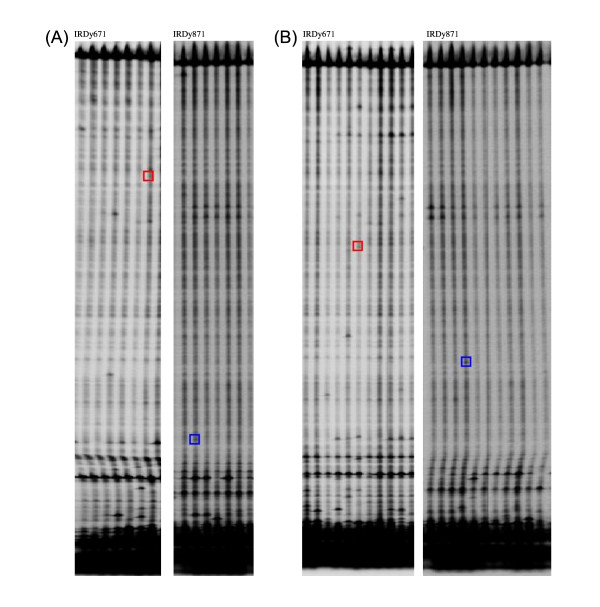
**Identification of mutant pools on LiCOR gel**. The two images represent different examples (A and B) of two identified mutant pools during the *kasII *gene screening; for each example, two images of the same gel electrophoresis are shown, respectively corresponding to the IRDy671 and IRDy781 fluorescence channels.

The *kasII *and *kasIII *genes encode two key enzymes of the fatty acid biosynthetic metabolism, the β-keto-acyl-ACP-synthetases II and III respectively. They mainly differ in their substrate specificities, in their sensitivity to some antibiotics, in the ability of the substrate to be activated by acyl carrier protein (ACP) or coenzyme A (CoA), and in the molecular mechanism of their reactions [34,35]. KASII is responsible for fatty acid chain elongation from 16 to 18 carbon atoms, showing highest activity towards the C_16_-ACP substrate. KASIII is the initial condensing enzyme catalysing the condensation of acetyl-CoA (C_2_-CoA) to malonil-ACP (C_4_-ACP) to produce 3-butyryl-ACP [36]. The complete coding sequence of the *kasII *gene (gi|112430752|gb|DQ835562.1|) and the EST sequence of *kasIII *gene (TC18876) were identified on NCBI and TGI databases and consisted respectively of 1,778 bp and 1,522 bp. Both sequences were subjected to CODDLE analysis to find the most suitable regions for the TILLING screen, i.e. regions where potentially deleterious lesions were most likely to be found. Since the genomic sequences were not available, a bioinformatic analysis of the putative gene structure and the level of conservation of both candidates were carried out. A set of primer pairs was then designed for each sequence with the aim of obtaining partially overlapping amplicons. After sequencing, the genomic sequence of each fragment allowed us to reconstruct the intron/exon model of the candidate genes, focusing in particular on the most promising regions. In this way, a region of ~700 bp for the *kasII *gene and one of ~1,500 bp for the *kasIII *gene were chosen for the pilot assay on the sub-set of M_2 _lines (Figure 4). The screening of 1,152 mutant lines for the *kasII *gene identified four mutants, confirmed by sequencing. All mutations identified were homozygous; three of them were localized to introns, while the mutation at position 714 of the sequence caused a G/T transversion, resulting in a premature stop-codon (E139*) (Table 4). Thus the mutation frequency of the *kasII *gene was estimated to be 1/168 kb. In the case of *kasIII *gene, 768 mutant lines were screened but no clear signals from mutated samples on the LiCOR gels were scored (Table 4).

**Table 4:** Pilot assay on a sub-set of M_2 _lines and estimation of the mutation frequencies.

Gene	Fragment size (bp)	Number of screened plants	Identified mutation					Mutation frequency*
				
			total	missense	stop codon	silent	intronic	
***kasII***	686	1152	4	0	1	0	3	1/168 kb
***kasIII***	1500	768	0	-	-	-	-	-
***fad2-1***	965	1152	3	1	-	1	1	1/332 kb
***Plasmopara***	1430	1152	2	-	-	-	2	1/766 kb

***Total***	4581		9					1/475 kb

* To calculate the mutation frequency, the fragment size of each gene was reduced to 100 bp because of priming and PCR artefacts.

Another important enzyme involved into the fatty acid pathway and responsible for the synthesis of linoleic acid from oleic acid is the isoform 1 of the Δ-12 oleate desaturase (FAD2; EC 1.3.1.35) encoded by the *fad2-1 *gene [37]. It is a membrane-bound enzyme that catalyzes the first extra-plastidial desaturation in plants, converting oleic acid to linoleic acid. The reaction involves the concomitant reduction of molecular oxygen to water and requires the presence of an electron donor system [38]. To identify the most suitable region for TILLING screening a 3,008 bp genomic sequence (EMBL: AY802989) was subjected to bioinformatic analysis. Based on the CODDLE prediction, a primer pair amplifying a 960 bp fragment was designed that flanked the unique large exon of the gene and used for screening. Three mutations were identified: one resulted in a missense change (F26L), a second caused a silent change (R46 =) and a third was situated in the non-coding region. The mutation frequency for this gene was estimated as 1/332 kb (Table 4).

The *AY490791 *gene is annotated on NCBI website as a LRR gene involved into *Plasmopara halstedii *resistance process. This biotrophic oomycete is the causal agent of downy mildew in wild and cultivated sunflower and over 100 host species in the *Asteraceae *family [39]. This is one of the main diseases in the cultivation of sunflower, causing economic losses of up to 95% [40]. The most promising region of this gene, a highly conserved 1,430 bp sequence, was selected. The molecular screening of 1,152 lines allowed us to identify two individuals with mutations in their intronic sequences, leading to an estimated mutation frequency for this gene of 1/766 kb.

## Discussion

Conventional mutagenesis using chemical and physical mutagens have been widely used as forward genetic approach for crop improvement, including sunflower species [29,41,42]. Mutagenic treatments have been successfully applied to improve oil-quality and to induce tolerance to biotic and abiotic stress, such as salt-, disease-, pest-, cold- and metal concentration-resistance [1]. New functional approaches, like RNA interference [43] or transposon mutagenesis [44], have been developed in the last decade, as a consequence of the rapid accumulation of large-scale sequence data and the increasing need to determine gene function for crop improvement. Several TILLING populations have been produced for different species, using EMS, MNU, diepoxybutane, NaN_3 _or a mixture of these chemicals as mutagenic agents [11,45]. Even though the sunflower genome has not been sequenced yet, a large number of EST sequences deriving from the Composite Genome Project and from several databases are available (http://compgenomics.ucdavis.edu/cgp_sitemap.php); (http://compbio.dfci.harvard.edu/tgi/plant.html); (http://www.heliagene.org). In the present study the first sunflower population for reverse genetic has been generated. To scan for mutations, fluorescent-labeling and double-stranded cutting of mismatches with the *Cel*I cleavage assay were combined with a gel electrophoresis-based analysis, according to Colbert *et al*. [17].

The identification of the optimal dose of a chemical mutagen that maximizes the mutation frequency without extreme toxic effects on the vitality and the fertility of biological tissues is a key factor during the establishment of a TILLING project. In fact, the main problem associated with chemical mutagenesis is the high cell toxicity of the mutagen, which can decrease the germination rate and viability of M_1 _seedlings to unacceptable levels without providing sufficient mutation density in the survival plants [11,46]. Moreover, in the next generation, undesirable phenotypes could appear as a result of the random distribution of mutations in the genome. This usually translates into the necessity to perform a preliminary 'kill curve' analysis, to find a compromise between mutagen toxicity, genome mutation saturation and possible accumulation of undesirable phenotypes [47,48].

Our aim was to assemble a sunflower TILLING population of fertile individuals, so that the progeny of each plant carrying a mutant allele could be directly recovered. To achieve this we initially performed a 'kill curve' analysis and then the most suitable EMS dosage was applied to our seed material without excessively compromising the M_1 _germination rate (~43%) and the M_2 _fertility rate (87%). Our data cannot be directly compared with those reported in other TILLING projects, since different negative effects to mutagenic treatment could be displayed according to the genetic background of the treated species or variety [17,45,49,50]. A sunflower TILLING population of 3,651 independent mutant lines was generated in this work, providing an important tool for the identification of interesting phenotypes.

Since considerable efforts are needed for the propagation of mutant lines to the second generation, a phenotypic marker of mutation frequency in the early stages of population development is often desirable. Thereby, seven phenotypic descriptors among those listed and promoted by the *International Board for Plant Genetic Resources *[13] were chosen for the phenotypic screening of our population. Moreover, because of the out-crossing nature of sunflower and the need to bag the inflorescences for self-pollination, the phenotypic scoring for flowering or flower morphology could not be performed. However, about 5% of the population showed altered morphologies in comparison with the wild-type. Some of the mutant lines (37) showed a single altered trait, while 138 lines displayed multiple mutant traits. Moreover, future screenings of the M_3 _generation will clarify the nature of the observed sunflower mutant lines by comparison of the phenotypic percentages recorded in two generations, as reported for indica rice IR64 [12] and tomato [51], where not all the identified M_2 _mutant lines were confirmed in the next generation.

Information about the genetic uniformity of both the untreated and mutagenized plant material and about the maintenance of seed stocks have been provided. This molecular marker analysis allowed us to detect the existence of any contamination of the population. The original seed stock represented an inbred line (F_8_), and two more selfing generations were performed to obtain the M_2 _progeny; thereby a high level of genetic uniformity and homozygosity was expected. However, out-crossing events with unwanted pollen may occur in field conditions, when an open pollinated species is analyzed and obliged to self-fertilize by bagging its inflorescences. The SSR genotyping performed in the present work confirmed the genetic uniformity and purity of both original seed stock and randomly analyzed M_2 _plants of the TILLING population. Wu *et al*. [12] reported this approach to be a cost-effective method of purity control of mutant stocks; they routinely genotyped the identified rice mutants to ensure they originated from the original seed stock, before seed distribution [12]. Moreover, SSR analysis allowed Xin *et al*. [52] to identify that 30% of M_2 _sorghum plants resulted from cross-pollination.

Several enzymes have been used for mismatching specific cleavage, but *Cel*I is the most common one in TILLING projects [11,45] for genotyping applications [53] and detection of heterozygous polymorphism [54]. The *Cel*I-mismatch cleavage assay allowed us to establish the best experimental conditions by the background reduction from gel images and the increase in SNP resolution power. The results obtained in our test provided a further advantage, since the use of minimal enzyme concentrations translated into a considerable economic saving.

Since DNA sample quality, normalization and pooling level can directly affect the efficiency and cost of mutation discovery [9,11], the main goal of our DNA pooling test was to maximize the throughput by increasing DNA pooling level while still clearly detecting the expected polymorphism. Although the simulation of a 20-fold pool of individual samples resulted in a successful detection of mutations, the 8-fold pool and the two-dimensional format were chosen as the pooling strategy for our sunflower TILLING population.

The TILLING strategy we developed has been successfully applied here as a pilot assay on 1,152 sunflower M_2 _lines. Four genes have been subjected to the reverse genetic screening: the *kasII *and *kasIII *genes, respectively codifying the isoforms II and III of the β-keto-acyl-ACP-synthetase; the *fad2-1 *gene, encoding the enzyme responsible of the converting reaction of oleic acid to linoleic acid; the *AY490791 *gene, involved in *P. halstedii *resistance. Our interest was first focused on some key enzymes of the fatty acid pathway, because of the interest in increasing the nutritional value of sunflower oil by the reduction of the ratio of saturated to unsaturated fatty acids. Moreover, *P. halstedii *is one of the most dangerous pathogens that affects sunflower cultivation in the Mediterranean area [39]. Therefore the availability of a stable and effective system, as genetic resistance, for the pest-control results of prime importance.

Since few genomic sequences are publicly available for sunflower, the reverse genetic screening was preceded by an accurate reconstruction of candidate gene models, by the amplification and the subsequent sequencing of short overlapping fragments. We focused our efforts in this first step on identifying for each candidate gene the most promising region for TILLING analysis. In this way, new primer pairs flanking this region could be targeted to the intronic sequences, with the aim of improving the screening efficiency on the coding regions in the pilot assay. In total, nine mutant lines have been identified. Each has been confirmed by sequencing and genotyped by microsatellite markers to exclude any individuals originating from cross-pollination events. The results of this first reverse genetic screening translated into an average mutation frequency of 1/475 kb, which is not substantially different from those observed in TILLING populations of other diploid species such as barley (1/374 kb for cv Morex and 1/500 kb for cv Barke) [55,56], *Arabidopsis *(1/300 kb) [10], *Brassica oleracea *(1/447 kb) [57], rice (1/280 kb for cv Nipponbare, 1/135 kb for cv Taichung 65 and 1/500 kb for cv IR64) [12,58,59] and sorghum (1/526 kb) [52].

## Conclusions

The establishment of the EMS-mutagenized TILLING population described here represents an important advance in the generation of new genetic variation in sunflower. The development of TILLING technology can lead to the identification of new alleles that may be directly of value for crop improvement. Furthermore, intensive investigation of the role of key genes, becomes a feasible goal, especially important where genomic information is lacking, as it is in sunflower. Moreover, TILLING methodology makes it possible to focus on specific genes or genomic regions, bypassing problems with other functional genomic tools (such as T-DNA knock-outs or RNAi-based gene silencing) that require the generation of transgenic plants. Thus we have developed and established an exciting tool for forward and reverse genetics in sunflower, one that is available for scientific collaborations and that aims to contribute to a global understanding of sunflower gene organization and regulation.

## Competing interests

The authors declare that they have no competing interests.

## Authors' contributions

WS set up and optimized the sunflower TILLING platform, organized the phenotypic database, carried out the microsatellite analysis, performed the molecular screening for the pilot test and wrote this manuscript. VA contributed to the M_1 _and M_2 _field trials and to the phenotypic screening of plant material. CM and AB conceived and coordinated the project and supervised the preparation of the manuscript. All authors read and approved the final manuscript.
